# Improving Sarcoma Outcomes: Target Trial Emulation to Compare the Impact of Unplanned and Planned Resections on the Outcome

**DOI:** 10.3390/cancers16132443

**Published:** 2024-07-03

**Authors:** Timothy T. A. F. Obergfell, Kim N. Nydegger, Philip Heesen, Georg Schelling, Beata Bode-Lesniewska, Gabriela Studer, Bruno Fuchs

**Affiliations:** 1 Faculty of Health Sciences and Medicine, University of Lucerne, Frohburgstrasse 3, 6002 Luzern, Switzerland; 2 Sarcoma Center, LUKS University Teaching Hospital, Luzerner Kantonsspital, 6000 Lucerne, Switzerland; georg.schelling@luks.ch (G.S.);; 3 Medizinische Fakultät, Universität Zürich, 8032 Zurich, Switzerland; 4 Sarkomzentrum KSW, Klinik für Orthopädie und Traumatologie, Kantonsspital Winterthur, 8400 Winterthur, Switzerland

**Keywords:** sarcoma, unplanned “whoops” resection, planned resection, Target Trial Emulation (TTE), local recurrence, metastasis-free survival, overall survival, multidisciplinary teams (MDT), benchmarking in healthcare, digital twin in medical research

## Abstract

**Simple Summary:**

In this study, we aimed to understand the association between two types of surgeries on sarcoma outcomes: surgeries that were planned with a clear understanding of the cancer (planned resections) and those that were performed unexpectedly, without prior knowledge that the tumor was cancerous (unplanned resections). Using the novel Target Trial Emulation framework, we assessed how these surgeries impact local recurrence-free survival, metastasis-free survival, cancer-specific survival, and overall survival. Our study found that patients who had unplanned surgeries were more likely to experience their cancer recur at the surgery site. However, there was not a substantial difference in how long patients survived after either type of surgery. Our findings highlight the importance of immediately referring patients to specialized sarcoma treatment centers where unplanned resections are less likely to occur to improve treatment outcomes and provide evidence to guide better management strategies for sarcoma.

**Abstract:**

This study follows the Target Trial Emulation (TTE) framework to assess the impact of unplanned resections (UEs) and planned resections (PEs) of sarcomas on local recurrence-free survival (LRFS), metastasis-free survival (MFS), cancer-specific survival (CSS), and overall survival (OS). Sarcomas, malignant tumors with mesenchymal differentiation, present a significant clinical challenge due to their rarity, complexity, and the frequent occurrence of UEs, which complicates effective management. Our analysis utilized real-world-time data from the Swiss Sarcoma Network, encompassing 429 patients, to compare the impact of UEs and PEs, adjusting for known prognostic factors through a multivariable Cox regression model and propensity score weighting. Our findings reveal a significantly higher risk of local recurrence for UEs and a short-term follow-up period that showed no marked differences in MFS, CSS, and OS between the UE and PE groups, underlining the importance of optimal initial surgical management. Furthermore, tumor grade was validated as a critical prognostic factor, influencing outcomes irrespective of surgical strategy. This study illuminates the need for improved referral systems to specialized sarcoma networks to prevent UEs and advocates for the integration of TTE in sarcoma research to enhance clinical guidelines and decision-making in sarcoma care. Future research should focus on the prospective validations of these findings and the exploration of integrated care models to reduce the incidence of UEs and improve patient outcomes.

## 1. Introduction

Sarcomas are malignant tumors distinguished by their mesenchymal differentiation [1] with an incidence of 5 cases per 100,000, classifying them as rare diseases [2]. The misdiagnosis and subsequent unplanned whoops resection (UE) of sarcomas, under the presumption of benignity, constitutes a significant clinical challenge, particularly in the case of smaller, superficial sarcomas in younger patients [3,4]. An average rate of 33% for UE among sarcoma patients, with observed rates ranging from 18% to 53%, was reported in a comprehensive review encompassing 33 studies and underscored the complexity and variability of sarcoma management practices [5]. However, an extensive study from Japan including a nationwide cancer registry with 8761 patients reported a rate of only 11% for UE [6]. The term UE itself encompasses a broad spectrum of scenarios in the literature, lacking a universally accepted (and standardized) definition. Studies have variously described UEs as the removal of a tumor without adequate preoperative diagnostics and consideration of surgical margins or any surgery conducted without specialized evaluation and planning based on the surgeon’s initial diagnosis. This diversity reflects the multifaceted nature of sarcoma diagnosis and the critical need for precise planning to avoid adverse outcomes [5,7,8,9].

The reasons for UE of sarcomas are manifold, primarily based on four key aspects. The overall rarity of sarcomas and, additionally, their subdivision into approximately 70 subgroups, each with distinct clinical and therapeutic differences; the intrinsic complexity of sarcomas, due to which, conventional diagnostic criteria for malignant tumors are not always directly applicable; the complex diagnostic process involving conventional macroscopy, immunohistochemistry, and molecular genetics, the latter requiring high professional expertise; and the significant disparity in expertise observed in rare diseases like sarcomas [2]. Another contributing factor may arise from the often-prolonged diagnostic intervals between the first physician visit and the engagement of an expert [10].

The impact on the clinical outcome following UE has not been conclusively studied. It is assumed that UEs are linked to reduced local recurrence-free survival (LRFS) compared to planned oncologic resections (PEs), while no disparity is evidenced concerning metastasis-free survival (MFS) and cancer-specific survival (CSS) [4,11,12,13]. Furthermore, there is an established correlation between residual tumor presence in the re-resected specimen and reduced LRFS, as well as overall survival (OS) [13,14,15,16,17]. Given the significant prevalence of residual tumors observed in 31–74% of cases following UE [5], it is reasonable to anticipate a reduction in LRFS and OS post-UE.

There is no uniform agreement on how to best approach patients after UE [18,19,20]. The UK guidelines recommend considering re-resection for UE with positive resection margins (R1/R2), highlighting the procedure’s potential to improve OS rates and extend MSFS [18,19,21]. Research, including a study by Nakamura et al., has shown similar survival and local recurrence rates between PE and UE, followed by subsequent re-resection [22]. Despite these benefits, corrective surgeries often require more complex reconstructive techniques, such as skin grafts, flaps, or even limb amputation [8], raising the critical question of whether re-resection should be universally applied to all UEs or only a select subset [20]. This unresolved dilemma highlights a significant gap in our current treatment paradigms, underscoring the pressing need for innovative research approaches that can elucidate and guide these crucial decisions in sarcoma care.

While retrospective studies certainly have provided valuable insights into the outcomes of surgical interventions for sarcomas, their inherent limitations, such as the inability to establish causality and control for confounding factors, have left critical questions unanswered. The gold standard of clinical studies, a randomized controlled trial (RCT), faces unsurmountable ethical hurdles when it comes to comparing UE versus PE, due to the direct impact on patient care and treatment decisions [23]. This critical juncture in sarcoma research underscores the urgent need for alternative methodologies that can offer the precision and reliability of RCTs without their ethical implications.

Target Trial Emulation (TTE) has emerged as a novel methodological framework that addresses these challenges [23,24,25]. By emulating the design principles of RCTs and utilizing real-world-time data, TTE allows for the ethical and rigorous exploration of treatment outcomes, effectively bypassing the ethical concerns tied to randomizing surgical treatment options [25]. Leveraging real-world-time data to approximate the causal inference of RCTs, TTE can also minimize potential selection biases typically associated with observational studies [25]. This innovative approach not only bridges the methodological divide but also opens up new avenues for exploring the nuanced impacts of surgical strategies on sarcoma patient outcomes. Adopting TTE, this represents a pivotal advancement in clinical research, enabling the investigation of UEs versus PEs with a level of scientific rigor previously deemed unattainable.

In brief, this study aims to assess the association between UE (compared to PE) and LRFS, MFS, CSS, and OS by emulating a target trial. By pioneering a methodological alternative to traditional RCT’s, we aim to shed new light on the implications of surgical strategies for sarcoma, potentially guiding more effective treatment pathways. This exploration promises to not only advance our understanding but also stimulate further research into the nuanced impacts of surgical decisions on patient outcomes in sarcoma care.

## 2. Materials and Methods

To uphold the quality in sarcoma care and to foster interdisciplinary exchanges, the Swiss Sarcoma Network (SSN) (https://www.swiss-sarcoma.net/, accessed on 31 May 2024) collects patient data longitudinally across the therapeutic continuum of soft tissues and bone tumors, creating a multi-center, high-quality real-world-time (RWT) data warehouse [26,27]. The current data repository, Adjumed.ch (Adjumed Services AG, Zurich, Switzerland; www.adjumed.ch, accessed on 10 October 2023), hosts the dataset, which is anticipated to transition to a new management system, Sarconnector^®^ (BF&PH, Zurich, Switzerland) to function as a dynamic real-world-time data (RWT) warehouse for automated analysis [28].

**Figure 1:**
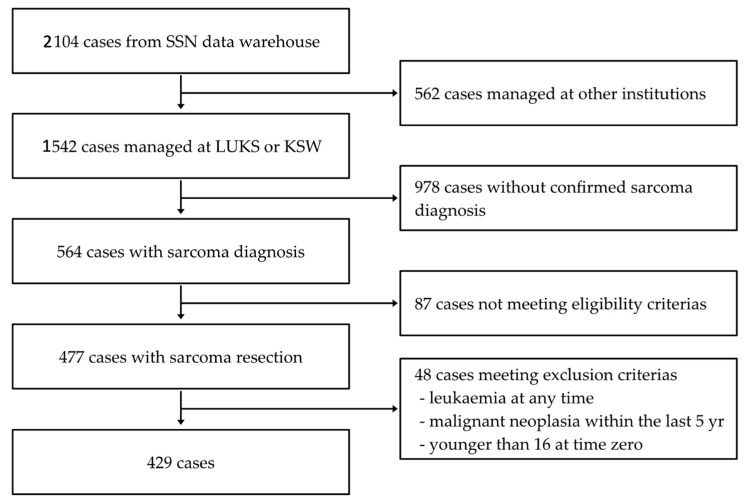
Flowchart.

The SSN data warehouse encompasses the data from 2104 patients registered by SSN members from 2018 to 2023, as illustrated in Figure 1. All sarcoma patients who underwent either UE or PE and received treatment (whether initial or follow-up) at the University Hospital Lucerne (LUKS) or the Cantonal Hospital Winterthur (KSW) were consecutively included (*n* = 477). In 87 cases, the inclusion criteria were not met: no index surgery was planned (*n* = 85), death occurred before the index surgery (*n* = 1), and no data on the index surgery were available (*n* = 1). Exclusion criteria were a history of any malignant neoplasm within the past 5 years (*n* = 36), any history of leukemia (*n* = 2), and age below 16 years (*n* = 10). Furthermore, desmoid tumors were not included in this study. The final cohort for analyzing the outcome of UE versus PE included 429 patients.

The observation period began at the point of the initial surgical intervention, either UE or PE, referred to as time zero, and ended with the occurrence of the predefined outcomes (local recurrence, metastasis, or death) or date of last patient contact. Due to the real-world-time structure of our data, time zero—the point of initial surgical intervention—can occur before the official start of the study inclusion period. This situation arises when a patient with a recurrence is enrolled in the study, potentially leading to an overestimation of the incidence of local recurrence in the presented cohort. Tumor reappearance at the original site was captured as local recurrence, tumor spread to a different site as metastasis, and mortality was subdivided into all-cause and death due to sarcoma. All consecutive patients were presented to the SSN-MDTB (Multidisciplinary Tumor Board) to determine their recommended individualized treatment strategy.

Utilizing the methodology of TTE, this study incorporated the principles of RCT to real-world-time data [23,25]. This approach involved the prior development of a target trial protocol (Table 1), creating a multivariable Cox regression model and implementing propensity score weighting to approximate the randomization process of a RCT. Hazard ratios (HRs) and 95% confidence intervals (CIs) are presented. The proportional hazards assumption in the Cox proportional hazards regression was verified using Schoenfeld residuals. The linearity assumption for the included covariates was checked using Martingale residuals. Regarding the heterogeneity of the study population, adjustments were made for known prognostic factors in a multivariable Cox regression model, including the tumor biological behavior, grade, size, and sarcoma classification, as well as the anatomical region of the tumor. Sarcoma classification is divided into axial (including head, neck, trunk, visceral, intraperitoneal, and retroperitoneal) and appendicular (encompassing upper and lower extremity). Biological behavior serves as an index for the inherent biological characteristics and clinical behavior, encompassing their growth pattern, invasiveness, metastatic potential, and potential response to treatment. These include three stages: benign, intermediate, and malignant, with benign soft tissue tumors not represented in this study. The propensity score was designed to equalize the distribution of baseline variables across the treatment arms—UE and PE—by being assigned in weights of the inverse probability of treatment assignment based on the aforementioned covariates.

To ensure the statistical analysis’s reliability, sensitivity analyses were performed, adjusting for additional variables such as age, sex, tumor volume (cm^3^), and resection status. The influence of chemotherapy and radiotherapy with curative intent on the outcome was also examined. Additionally, the subsets of patients without atypical lipomatous tumor (ALT) and dermatofibrosarcoma protuberans (DFSP) were analyzed. Because 76 cases had time zero prior to 2018 and were partly subsequently captured, an additional sensitivity analysis was designed to include only cases with time zero from 2018 onwards (See Appendix A Table A1, Table A2, Table A3 and Table A4).

Kaplan–Meier curves were employed to visually compare the time-to-event data between the UE and PE groups.

Statistical analysis was done using R statistical software (Posit, PBC, Boston, MA, USA, Version 4.3.2) [29]. A *p*-value < 0.05 was considered statistically significant.

## 3. Results

This study included 429 patients: 106 (24.7%) underwent UE and 323 (75.3%) underwent PE. The patient characteristics are presented in Table 2 and Table 3. The median follow-up time was 1.9 years (1st quartile (Q1), 3rd quartile (Q3); 0.8 years, 3.7 years). In the UE group, 58.4% had a reoperation, with a residual tumor present in resectate in 75.8% after reoperation. In nine cases, achieving R0 status during reoperation was not feasible due to local circumstances, and among these cases, six subsequently developed a local recurrence. Of the patients who underwent UE, 25.5% had reoperation alone, 33.0% received reoperation along with radiotherapy within 6 months, and 6.6% were treated with radiotherapy alone within 6 months. UE sarcomas were generally smaller (median 46.5 mm versus 94 mm), more frequently located superficially (35.8% versus 9.6%), and were often of lower grade (47.5% versus 30.2%). Notably, patients with dedifferentiated liposarcoma more commonly underwent PE (3.8% versus 11.1%).

### 3.1. Impact on Local Recurrence-Free Survival

**Figure 2:**
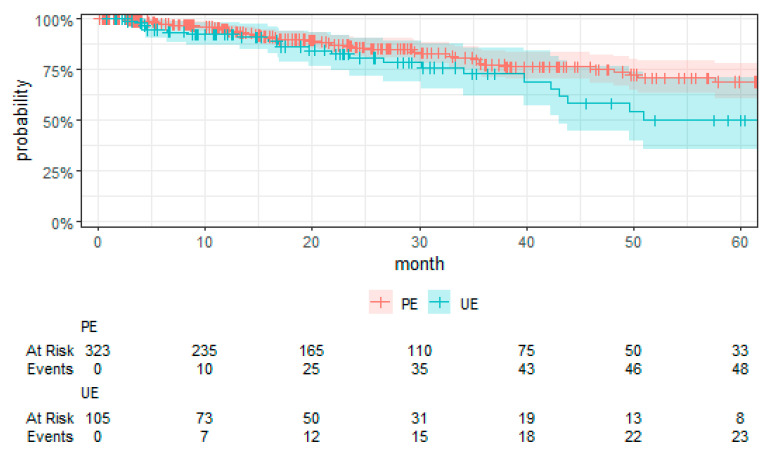
Kaplan–Meier plot of LRFS with the 95% confidence interval.

An analysis of the association between UE and LRFS, as shown in Table 4, indicated that patients with UE had significantly higher hazards of local recurrence (HR 7.49 (2.88, 19.49), *p* < 0.001) compared to patients that underwent PE. In this regard, the sensitivity analysis (see Appendix A Table A1) revealed no impact on the significance. Figure 2 presents the Kaplan–Meier curve for LRFS by treatment group. Grades 2 and 3 compared to grade 1 were significantly associated with local recurrence, HR 8.03 (1.09, 59.04), *p* = 0.04 and HR 8.50 (1.21, 59.48), *p* = 0.03, respectively. Biological behavior, sarcoma classification, grade, and anatomical region did not significantly influence the local recurrence risk.

### 3.2. Impact on Metastasis-Free Survival

**Figure 3:**
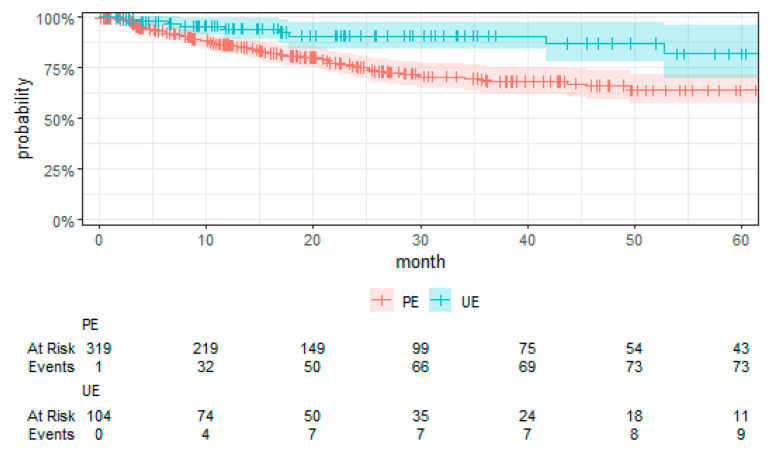
Kaplan–Meier plot of MFS with the 95% confidence interval.

The examination of UE’s association with MFS revealed no significant difference in MFS when compared to PE (HR 1.42 (0.40, 4.98), *p* = 0.58) (See Table 5). The sensitivity analysis (see Appendix A Table A2) revealed no impact on this result. Figure 3 presents the Kaplan–Meier curve for MFS by treatment group. Bone sarcomas had a higher hazard of metastasis compared to SST (HR 4.40 (1.29, 15.02), *p* = 0.02), with a similar trend observed for DST (HR 2.51 (0.85, 7.35), *p* = 0.09). High-grade sarcomas (grade 3) demonstrated a substantially higher hazard of metastasis compared to low-grade sarcomas (grade 1) (HR 11.20 (1.32, 95.20), *p* = 0.03), as did grade 3 compared to grade 2 (HR 3.87 (1.68, 8.94), *p* < 0.001).

### 3.3. Impact on Survival

#### 3.3.1. Overall Survival

**Figure 4:**
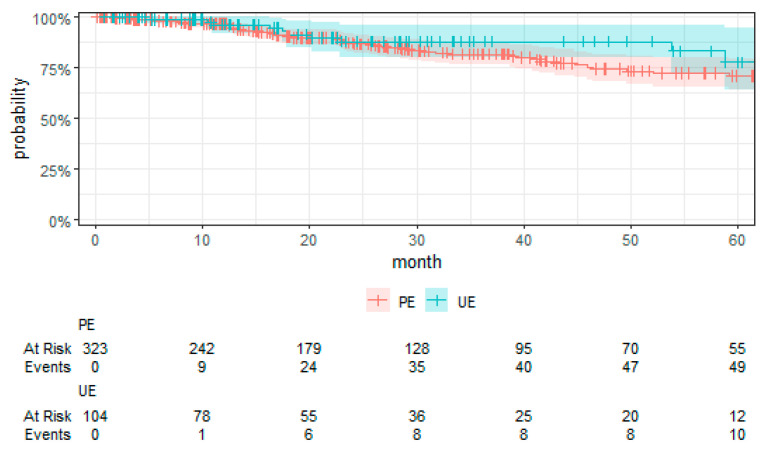
Kaplan–Meier plot of OS with the 95% confidence interval.

The hazards of death from any cause were not significantly increased after UE compared to PE (HR 1.47 (0.42, 5.16), *p* = 0.55) (Table 6). Furthermore, the sensitivity analysis (see Appendix A Table A3) did not lead to a substantially different result. The Kaplan–Meier survival curve for OS is displayed in Figure 4. However, the histological grade was an independent prognostic factor, sarcomas with grade 3 versus grade 1 (HR 29.00 (3.34, 251.99), *p* = 0.002) and grade 3 versus grade 2 (HR 12.82 (4.17, 39.36), *p* < 0.001) showing an increased risk of death. Biological behavior, sarcoma classification, anatomical region, and largest tumor diameter were not significantly associated with OS.

#### 3.3.2. Cancer-Specific Survival

**Figure 5:**
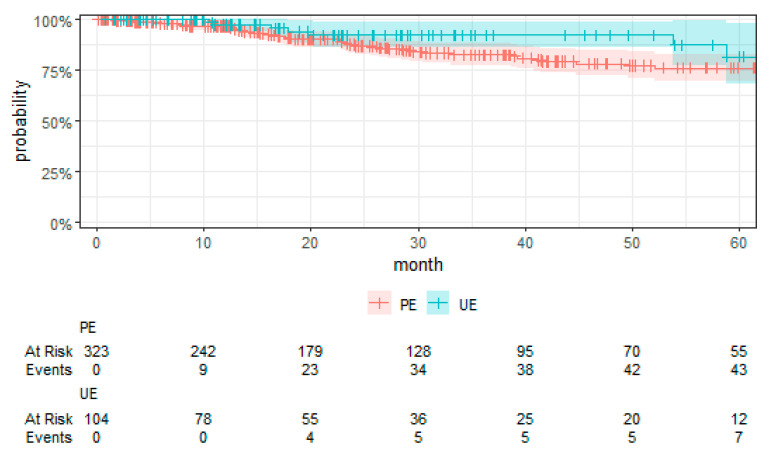
Kaplan–Meier plot of CSS with the 95% confidence interval.

The risks of death from sarcomas were not increased after UE compared to PE (HR 1.47 (0.37, 5.94), *p* = 0.59) (Table 7). The sensitivity analysis (see Appendix A Table A4) revealed no impact on the absence of significance. Figure 5 presents the Kaplan–Meier curve for CSS by treatment group. Yet, a higher risk of cancer-specific death was noted with G3 sarcomas compared to G1 (HR 39.94 (2.77, 575.65), *p* = 0.01) or G2 (HR 13.48 (4.15, 43.87), *p* < 0.001). Biological behavior, sarcoma classification, anatomical region, and largest tumor diameter were not significantly associated with CSS outcomes.

## 4. Discussion

For the first time, employing TTE to overcome the limitations associated with RCTs, this study presents a comprehensive evaluation of surgical strategies for sarcoma treatment. Our analysis revealed a notably higher risk of local recurrence in patients undergoing UEs compared to those receiving PEs, with a hazard ratio that underscores the significance of the initial surgical management on disease control. We found no significant differences in MFS and OS between the UE and PE groups. These findings suggest that, despite the adverse impact of UEs on local control, subsequent therapeutic interventions might mitigate the short-term risks of metastasis and mortality. Moreover, the tumor grade emerged as a significant prognostic factor, affirming its established role in influencing sarcoma outcomes. High-grade tumors were associated with increased risks of local recurrence, metastasis, and death, highlighting the critical importance of tumor biology in patient prognosis. These key findings contribute to the ongoing discourse on optimal sarcoma management strategies, emphasizing the need for precision in surgical planning and the potential benefits of incorporating TTE into oncological research.

Our study elucidates the complex interplay between surgical strategies and sarcoma outcomes, revealing a notably higher risk of local recurrence for patients undergoing UEs compared to PEs. This finding aligns with the literature but notably is in the upper end of the typical 5-year reported local recurrence rates (8% to 17%), pointing to a potential for non-referral selection bias in our RWT cohort [4,11,12,13,30,31,32,33,34]. Despite these concerns, our use of TTE effectively navigates these challenges by approximating the rigor of RCTs, providing a robust framework for comparing UE and PE outcomes despite inherent limitations such as selection bias [24,25,35].

Contrary to conventional expectations, our analysis found no significant differences in MFS, CSS, and OS between the UE and PE groups. This deviation from the expected outcomes suggests that subsequent therapeutic interventions may mitigate the adverse impact of UEs on short-term outcomes. The role of tumor grade as a prognostic factor further complicates the relationship between surgical strategy and sarcoma outcomes, with high-grade tumors associated with increased risks irrespective of the initial surgical approach [36,37,38].

The observed variations in the follow-up treatments for UE patients reflect the individualized nature of sarcoma management post-surgery [8,9,18,20,33]. This diversity in treatment strategies, alongside the differential outcomes reported, underscores the importance of considering both biological aggressiveness and post-surgical care in patient prognosis and treatment planning [36,37,38,39,40,41].

In light of our findings, the elevated risk of local recurrence post-UE and the nuanced interplay between surgical strategy and tumor biology underscore the need for refined surgical guidelines and patient management strategies in sarcoma care. These insights, alongside the observed disparities in the existing literature, underscore the critical areas for future research, particularly in exploring how individualized treatment strategies post-surgery can influence short-term outcomes. By highlighting these novel aspects, our study points to the significance of integrating the multidisciplinary network approach into the decision-making process, urging a shift towards more personalized and evidence-based approaches in sarcoma treatment planning.

Employing TTE has enabled us to navigate the ethical and logistical hurdles that preclude RCTs in sarcoma research, allowing for a rigorous comparison of UE and PE. This study’s insights highlight TTE’s utility in generating evidence-based recommendations for sarcoma management, particularly by enhancing our understanding of the differential impacts of surgical strategies [23,24,25]. Our findings advocate for TTE’s broader application, potentially enriching clinical guidelines and decision-making processes in sarcoma care. Using this methodology, we have demonstrated not just a way to address observational studies’ methodological challenges in sarcoma research but also a means to propel sarcoma research towards more personalized and evidence-based treatment strategies, aligning with findings from the existing literature [3,4,5,6] and setting a precedent for future studies to follow.

While our study leverages the prospective collection of data within the SSN to enhance the robustness of our findings, the retrospective analysis of these data introduces inherent limitations. Notably, including patients diagnosed before 2018 who later presented with recurrences may augment the observed rate of local recurrence, reflecting a form of non-referral selection bias. This aspect necessitates cautious interpretation of our results, particularly concerning the generalizability of our findings across all sarcoma subtypes and treatment modalities. Furthermore, despite the methodological rigor provided by TTE, challenges such as selection bias and the complete balancing of cohort characteristics remain. Additionally, with a median follow-up of 1.9 years, statements about long-term prognoses are hardly possible. These limitations highlight the need for future prospective studies to validate our findings and explore the comprehensive impact of surgical strategies on sarcoma outcomes in a more controlled setting.

The significant risk of local recurrence following UE highlights the critical need for rigorous surgical margin evaluation and, when necessary, individualized re-resection based on data evidence [18,19,20,21,33]. This emphasizes the essential role of a multidisciplinary approach in sarcoma management, where decisions are informed by a comprehensive understanding of tumor biology, potentially enhanced by our application of TTE. TTE’s ability to simulate RCT-like conditions provides a robust basis for refining treatment guidelines, facilitating personalized care that integrates tumor-specific biological characteristics [23,24,25].

Looking forward, it is imperative that future research undertakes prospective studies to validate our findings, with a particular focus on the role of tumor biology in determining treatment efficacy and patient outcomes [36,37,38,39,40,41]. Additionally, examining how surgical choices affect patient quality of life will be crucial. Such studies should extend beyond traditional survival metrics to capture the broader impact of treatment strategies on the well-being of sarcoma patients, aligning with the shift towards patient-centered care in oncology [8,9,18,33]. This comprehensive approach promises not only to enhance clinical outcomes but also to improve the overall quality of life for individuals facing sarcomas, advocating for treatment plans that are as unique as the patients themselves [20,22].

Ultimately, our study calls for an integration of clinical expertise and multidisciplinary perspectives in developing evidence-based, patient-specific strategies for sarcoma treatment. By embracing the insights provided by TTE and prioritizing the exploration of tumor biology alongside patient-centric considerations, we can advance towards more effective and nuanced care for sarcoma patients.

## 5. Conclusions

Our application of TTE highlights the significant risk of local recurrence stemming from UE in sarcoma care, illuminating a crucial systemic flaw in the referral to specialized sarcoma networks. This revelation underscores the urgent need for improved collaboration and integration across healthcare systems to ensure that all sarcoma patients receive care coordinated by multidisciplinary teams within specialized networks from the beginning. By fostering stronger links and clearer pathways for referral to sarcoma centers (Hub and Spoke Model), we can move towards a more unified and evidence-based approach to sarcoma management. Future directions must prioritize prospective studies to explore the effectiveness of these integrated care models in reducing the incidence of UE and enhancing overall patient outcomes. This also highlights the need for comprehensive and well-maintained cancer registries.

## Figures and Tables

**Table 1:** Target trial protocol—based on Edouard L. Fu 2023 [25].

Protocol Element	Target Trial	Emulation with Observational Data from the LUKS/KSW
Eligibility criteria	Patients which had a surgery with curative intent due to any kind of sarcoma. Sarcoma could be diagnosed before or due to this resection.	Same as target trial
Exclusion criteria	All Patients who met the following exclusion criteria: malignant neoplasia within the last 5 years any leukemia at any time in patient’s medical history younger than 16 at time zero	Same as target trial
Treatment strategies	1. Planned sarcoma resection 2. Unplanned Whoops resection	Same as target trial
Treatment assignment	Randomization, double-blinded	Eligible individuals are assigned at baseline to treatment strategy that their data are consistent with. To emulate randomization, we evaluated the probability of each patient being assigned to their group and adjusted for the following baseline variables: Biological behavior, sarcoma classification (SST, DST, bone *), anatomic region, tumor size, and grade.
Outcomes	Primary outcome: LRFS Secondary outcomes: MFS, CSS, and OS	Same as target trial
Causal estimation	The effect of UE on MFS, LRFS, CSS, and OS.	Same as target trial
Start and end of follow-up	Starts at the first surgical intervention after the randomization. Ends at occurrence of end point, administrative censoring, or 5 years of follow-up.	Starts at time zero (first surgery due to sarcoma regardless of whether the first surgery was a UE or a PE). Ends at occurrence of end point or administrative censoring.
Statistical analysis	Intention-to-treat analysis, non-naïve per protocol analysis	TTE with multivariable Cox regression and propensity scores

* Including osteosarcomas and Ewing sarcomas.

**Table 2:** Characteristics of the study cohort stratified by treatment group.

	All	PE	UE	*p*-Value
*n*	429	323	106	-
Gender				
Male, *n* (%)	229 (53.4)	167 (51.7)	62 (58,5)	0.26
Female, *n* (%)	200 (46.6)	156 (48.3)	44 (41.5)	0.26
Age in years, median (Q1, Q3)	63 (51, 74)	63 (53, 74)	63.5 (47, 75)	0.45
Sarcoma classification				
SST, *n* (%)	69 (16.1)	31 (9.6)	38 (35.8)	<0.001
DST, *n* (%)	317 (73.9)	256 (79.3)	61 (57.5)	<0.001
Bone, *n* (%)	43 (10.0)	36 (11.1)	7 (6.6)	0.20
Diagnosis *				
Myxoid liposarcoma, *n* (%)	28 (6.5)	24 (7.4)	4 (3.8)	0.26
Dedifferentiated liposarcoma, *n* (%)	40 (9.3)	36 (11.1)	4 (3.8)	0.02
Leiomyosarcoma, *n* (%)	47 (11.0)	31 (9.6)	16 (15.1)	0.15
Atypical lipomatous tumor	63 (14.7)	51 (15.8)	12 (11.3)	0.34
Undifferentiated/unclassified sarcoma	64 (14.9)	52 (15.8)	12 (11.3)	0.27
Tumor size (largest diameter) in mm, median (Q1, Q3)	80 (50, 129)	94 (60, 143)	46.5 (27, 79)	<0.001
Anatomic region				
Axial, *n* (%)	201 (46.9)	152 (47.1)	49 (46.2)	0.91
Appendicular, *n* (%)	228 (53.1)	171 (52.9)	57 (53.8)	0.91
Biological behaviour				
Intermediate, *n* (%)	112 (26.1)	78 (24.2)	34 (32.1)	0.13
Malignant, *n* (%)	317 (73.9)	245 (75.9)	72 (67.9)	0.13
Grade ^§^ (time zero)				
1, *n* (%)	138 (34.5)	91 (30.2)	47 (47.5)	0.004
2, *n* (%)	73 (18.3)	58 (19.3)	15 (15.2)	0.46
3, *n* (%)	189 (47.3)	152 (50.5)	37 (37.4)	0.03
Resection status (time zero) ^†^				
R0, *n* (%)	253 (63.1)	241 (78.8)	12 (12.6)	<0.001
R1/R2, *n* (%)	148 (36.9)	65 (21.2)	83 (87.4)	<0.001
Local recurrence, *n* (%)	82 (19.1)	56 (17.3)	26 (24.5)	0.27
Metastasis at diagnosis, *n* (%)	23 (5.4)	20 (6.2)	3 (2.8)	0.22
Metastasis at follow-up, *n* (%)	111 (25.9)	100 (31.0)	11 (10.4)	<0.001
Death				
Overall, *n* (%)	71 (16.6)	61 (18.9)	10 (9.4)	0.25
Cancer-specific, *n* (%)	61 (14.2)	54 (16.7)	7 (6.6)	0.01

The percentages relate to the *n*-values listed in the first row of the respective columns. * Only 5 common diagnoses in our study cohort are listed. ^§^ The grade corresponds to the FNCLCC system. ^†^ R0 status is determined by histological examination. Unlike R2, R1 status is characterized by a negative postoperative MRI finding. To distinguish between R1 and R2 statuses, the surgical report was also reviewed.

**Table 3:** Characteristics of the UE group.

	*n* (%)
Reoperation	62 (58.4)
Resection status (reoperation)	
R0	51 (48.1)
R1	8 (7.5)
R2	1 (0.9)
Tumor residual in the resectate after reoperation (histopathological)	44 (75.8) *
Reoperation with radiotherapy ^†^	27 (25.5)
Reoperation only	35 (33.0)
Radiotherapy only	7 (6.6)
Reoperation	62 (58.4)

* Out of the 62 reoperations after UE, only 58 histopathological reports were available. The percentages are based on these 58 histopathological reports. ^†^ Radiotherapy initiated within the first 6 months following UE only.

**Table 4:** Multivariable propensity score weighted Cox regression for LRFS.

Characteristics	HR	95% CI	*p*-Value
UE	7.49	2.88, 19.49	<0.001
Biological Behavior			
Intermediate	—	—	
Malignant	1.75	0.26, 11.67	0.56
Sarcoma classification			
SST	—	—	
DST	1.44	0.50, 4.16	0.50
Bone	1.06	0.24, 4.70	0.93
Bone (versus DST) *	0.74	0.23, 2.37	0.61
Anatomic region			
Appendicular	—	—	
Axial	1.96	0.80, 4.77	0.14
Largest tumor diameter (mm)	1.00	1.00, 1.01	0.03
Grade			
1	—	—	
2	8.03	1.09, 59.04	0.04
3	8.50	1.21, 59.48	0.03
3 (versus 2) *	1.06	0.56, 1.99	0.86

* After releveling, bone was compared to DST, and grade 3 was compared to grade 2.

**Table 5:** Multivariable propensity score weighted Cox regression for MFS.

Characteristics	HR	95% CI	*p*-Value
UE	1.42	0.40, 4.98	0.58
Biological Behavior			
Intermediate	—	—	
Malignant	1.24	0.08, 18.72	0.88
Sarcoma classification			
SST	—	—	
DST	2.51	0.85, 7.35	0.09
Bone	4.40	1.29, 15.02	0.02
Bone (versus DST) *	1.75	0.89, 3.47	0.11
Anatomic region			
Appendicular	—	—	
Axial	1.21	0.63, 2.30	0.57
Largest tumor diameter (mm)	1.00	0.99, 1.00	0.24
Grade			
1	—	—	
2	2.89	0.25, 34.07	0.40
3	11.20	1.32, 95.20	0.03
3 (versus 2)	3.87	1.68, 8.94	<0.001

* After releveling, bone was compared to DST, and grade 3 was compared to grade 2.

**Table 6:** Multivariable propensity score weighted Cox regression for OS.

Characteristics	HR	95% CI	*p*-Value
UE	1.47	0.42, 5.16	0.55
Biological Behavior			
Intermediate	—	—	
Malignant	0.77	0.12, 4.91	0.79
Sarcoma classification			
SST	—	—	
DST	2.58	0.58, 11.51	0.21
Bone	2.05	0.35, 11.99	0.43
Bone (versus DST) *	0.79	0.32, 1.98	0.62
Anatomic region			
Appendicular	—	—	
Axial	0.49	0.19, 1.24	0.13
Largest tumor diameter (mm)	1.00	1.00, 1.01	0.24
Grade			
1	—	—	
2	2.26	0.23, 21.90	0.48
3	29.00	3.34, 251.99	0.002
3 (versus 2)	12.82	4.17, 39.36	<0.001

* After releveling, bone was compared to DST, and grade 3 was compared to grade 2.

**Table 7:** Multivariable propensity score weighted Cox regression for CSS.

Characteristics	HR	95% CI	*p*-Value
UE	1.47	0.37, 5.94	0.59
Biological Behavior			
Intermediate	—	—	
Malignant	0.80	0.09, 7.01	0.84
Sarcoma classification			
SST	—	—	
DST	2.54	0.49, 13.12	0.27
Bone	2.37	0.35, 16.02	0.38
Bone (versus DST) *	0.93	0.36, 2.39	0.89
Anatomic region			
Appendicular	—	—	
Axial	0.50	0.18, 1.39	0.18
Largest tumor diameter (mm)	1.00	1.00, 1.01	0.22
Grade			
1	—	—	
2	2.96	0.19, 46.56	0.44
3	39.94	2.77, 575.65	0.01
3 (versus 2)	13.48	4.15, 43.87	<0.001

* After releveling, bone was compared to DST, and grade 3 was compared to grade 2.

## Data Availability

The data presented in this study are available on request from the corresponding author.

## Appendix A

**Table A1:** Sensitivity analysis: impact of UE on LRFS with additional characteristics and exclusions, respectively.

	HR	95% CI	*p*-Value
Additional Characteristics			
Age *	7.48	2.92, 19.2	<0.001
Resection status (R0, R1/R2) *	2.89	1.05, 7.98	0.04
Tumor volume (cm^3^) ^†^	6.51	2.75, 15.42	<0.001
Sex (female, male) *	7.27	2.87, 18.42	<0.001
Chemotherapy with curative intention *	10.68	3.97, 28.73	<0.001
Radiotherapy with curative intention *	6.60	2.71, 16.09	<0.001
Additional exclusion criteria			
ALT and DFSP	7.08	2.67, 18.73	<0.001
Cases with time zero before 1 January 2018	8.11	2.69, 24.41	<0.001

* Instead of the characteristic “biological behavior”. ^†^ Instead of the characteristic “largest tumor diameter (mm)”.

**Table A2:** Sensitivity analysis: impact of UE on MFS with additional characteristics and exclusions, respectively.

	HR	95% CI	*p*-Value
Additional Characteristics			
Age *	1.38	0.39, 4.93	0.62
Resection status (R0, R1/R2) *	0.79	0.24, 2.62	0.70
Tumor volume (cm^3^) ^†^	1.57	0.44, 5.64	0.49
Sex (female, male) *	1.26	0.36, 4.37	0.72
Chemotherapy with curative intention *	1.17	0.35, 3.91	0.79
Radiotherapy with curative intention *	1.32	0.42, 4.16	0.63
Additional exclusion criteria			
ALT and DFSP	1.40	0.40, 4.87	0.60
Cases with time zero before 1 January 2018	1.40	0.30, 6.44	0.67

* Instead of the characteristic “biological behavior”. ^†^ Instead of the characteristic “largest tumor diameter (mm)”.

**Table A3:** Sensitivity analysis: impact of UE on OS with additional characteristics and exclusions, respectively.

	HR	95% CI	*p*-Value
Additional Characteristics			
Age *	1.32	0.42, 4.20	0.64
Resection status (R0, R1/R2) *	2.19	0.62, 7.72	0.22
Tumor volume (cm^3^) ^†^	2.06	0.70, 6.08	0.19
Sex (female, male) *	1.54	0.55, 4.32	0.41
Chemotherapy with curative intention *	1.17	0.28, 4.87	0.83
Radiotherapy with curative intention *	1.49	0.50, 4.38	0.47
Additional exclusion criteria			
ALT and DFSP	1.53	0.47, 4.99	0.48
Cases with time zero before 1 January 2018	1.30	0.26, 6.43	0.74

* Instead of the characteristic “biological behavior”. ^†^ Instead of the characteristic “largest tumor diameter (mm)”.

**Table A4:** Sensitivity analysis: impact of UE on CSS with additional characteristics and exclusions respectively.

	HR	95% CI	*p*-Value
Additional Characteristics			
Age *	1.36	0.36, 5.15	0.60
Resection status (R0, R1/R2) *	0.92	0.31, 2.75	0.89
Tumor volume (cm^3^) ^†^	2.23	0.74, 6.76	0.15
Sex (female, male) *	1.53	0.50, 4.69	0.46
Chemotherapy with curative intention *	1.21	0.27, 5.49	0.81
Radiotherapy with curative intention *	1.57	0.49, 5.06	0.45
Additional exclusion criteria			
ALT and DFSP	1.54	0.42, 5.72	0.52
Cases with time zero before 1 January 2018	1.25	0.21, 7.33	0.81

* Instead of the characteristic “biological behavior”. ^†^ Instead of the characteristic “largest tumor diameter (mm)”.
